# Exploring the optimal age for total knee arthroplasty to minimize risk of adverse outcomes: machine learning analysis of a statewide cohort

**DOI:** 10.1186/s42836-026-00372-z

**Published:** 2026-02-28

**Authors:** Chloe Heiting, Yiyuan Wu, Susan M. Goodman, Peter Sculco, Fei Wang, Said Ibrahim, Peter Cram, Rich Caruana, Bella Mehta

**Affiliations:** 1 Donald and Barbara Zucker School of Medicine at Hofstra/Northwell, Hempstead, NY 11549 USA; 2 Weill Cornell Medicine, New York, NY 10065 USA; 3 Hospital for Special Surgery, New York, NY 10021 USA; 4 Sidney Kimmel Medical College, Philadelphia, PA 19107 USA; 5 University of Maryland School of Medicine, Baltimore, MD 21201 USA; 6 Intelligible Inc., Redmond, WA 98052 USA

**Keywords:** Age, Risk assessment, Total knee arthroplasty, Outcomes, Machine learning, Artificial intelligence

## Abstract

**Background:**

Rates of total knee arthroplasty (TKA) in the United States have risen in patients of a wide age range. Although rates of postoperative TKA complications have decreased, they remain a significant concern. In this study, we aim to determine how the risk of adverse TKA outcomes changes dynamically with age and explore the optimal ages with the lowest risk for adverse outcomes.

**Methods:**

This retrospective cohort study included patients who underwent elective primary TKA from 2012 to 2018 in the Pennsylvania Health Care Cost Containment Council Database. We trained (70% train:30% test) an explainable boosting machine (EBM), a modern generalized additive model, to predict risk for 90-day mortality, 90-day readmission, 1-year revision, and longer length of stay (LOS). This “glass box” model allowed us to measure and visualize feature importance using mean absolute scores and determine the role of age in the model. We then ran EBM models that allowed two-way interactions between age and patient-level covariates.

**Results:**

In our cohort of 227,959 patients, 90-day readmission was observed in 7.5%, 90-day mortality in 0.2%, and 1-year revision in 0.8%. The median LOS was 2 days (IQR [2, 3]). Age was among the most important factors for predicting all outcomes, and these were nonlinear relationships. The risk for 90-day mortality increased substantially at 76.5 years, and for 90-day readmission and longer LOS at 73.5 years. Risk for 1-year revision was greater before 63.5 years.

**Conclusions:**

We determined that there is a nonlinear relationship between age and risk for adverse TKA outcomes, and it changes dramatically at specific time points. Our data suggests that the optimal age for lower risk of 90-day mortality, 90-day readmission, and longer LOS is below 73.5 years, and above 63.5 years for 1-year revision. These findings can help in decision-making when trying to quantify risks related to aging.

## Introduction

Total knee arthroplasty (TKA) is a typically elective procedure performed to relieve pain and improve function and quality of life in patients with osteoarthritis (OA). TKA use in the United States (US) has risen [1–4], in part due to the aging population, as well as a rise in obesity and OA at a younger age [5]. Although the average age for TKA in the US was 67 years in 2021 [6], data suggests there is an increasing demand for TKAs in younger patients [4, 7, 8].

Postoperative complications in TKA have decreased with advancements in surgical techniques, implant design, and perioperative care, yet some complications still occur today [9, 10]. Age remains an important factor in TKA outcomes. It is known that mortality increases with age [9, 11, 12], and there are greater rates of TKA revision in younger patients [13–15].

Most studies examining risk factors for TKA outcomes use traditional regression models and provide an odds ratio, where an age or age range is associated with an increased or decreased risk of poor outcome. However, the relationships between age and adverse TKA outcomes may be more complex and are often nonlinear. Understanding this relationship may inform patient and provider planning and decision of TKA. In this study, we aimed to use a glass box machine learning model to address the following questions: (1) How does the risk of adverse TKA outcomes change with age? (2) What are the optimal ages with the lowest risk for adverse outcomes?

## Methods

### Data source

We analyzed retrospective data from the Pennsylvania Health Care Cost Containment Council (PHC4) Database from 2012–2018. The PHC4 dataset contains over 70 fields for more than 4.5 million procedures performed in 170 Pennsylvania Assigned Facilities or non-governmental acute care hospitals in Pennsylvania [16]. This study was IRB-exempt.

### Study cohort

This retrospective study included patients who underwent elective primary TKA using *International Classification of Diseases*, *Ninth Revision*, *Clinical Modification* (ICD-9-CM) codes from 2012 through September 2015, and *ICD*, *Tenth Revision*(ICD-10) codes from October 2015 through 2018. The ICD-9-CM procedure code 81.54 and ICD-10 codes 0SRC0xx or 0SRD0xx were used, which are validated codes from the American Joint Replacement registry with a sensitivity of 89%, specificity of 98%, and a positive predictive value of 97% [17, 18]. The study cohort and methodology have previously been described [19].

A total of 258,724 patients were identified, and 30,765 patients were excluded. Exclusion criteria were patients with: (1) diagnostic codes suggesting inflammatory arthritis (e.g., rheumatoid arthritis), pathologic fracture, avascular necrosis, and metastatic and bone cancer; (2) non-elective admissions or inter-hospital transfers prior to TKA; (3) ≥ 2 past TKAs; (4) bilateral TKA during the index hospitalization; (5) revision during the index hospitalization; (6) missing key data variables such as age and sex; and (7) age < 18 years or ≥ 105 years.

We extracted information on patient-level covariates from the PHC4 dataset, including age, sex, race, discharge location, admission source, and insurance. We identified medical comorbidities with the Elixhauser Comorbidity index developed by Quan et al [20]. We also separately included diabetes, hypertension, and obesity since they are known to increase adverse outcomes in TKA [21–23].

### Statistical analyses

We described patient-level demographics for those undergoing TKA using N (%) for categorical variables, and median [IQR] or mean (SD) for continuous variables. Differences in patient-level demographics, comorbidities, and hospitalization characteristics were compared based on 90-day readmission, 90-day mortality, and 1-year revision. We used Pearson’s *Chi*-squared and/or Fisher’s exact tests for categorical variables, and the Wilcoxon rank sum test for continuous variables. Length of stay (LOS) was log transformed (equation log(LOS + 0.5)) to alleviate issues with skewed distribution and LOS being 0.

We trained a supervised machine learning model—explainable boosting machine (EBM)—with a 70% training and 30% testing split on data stratified by each outcome to predict risk for 90-day mortality, 90-day readmission, 1-year revision, and LOS. The model included demographics, comorbidities, insurance, and other admission-related characteristics. EBMs are a modern form of generalized additive models characterized by full-interpretability and prediction accuracy comparable to random forests and other tree-based machine learning models [24, 25]. These models are thus often referred to as “glass box” models as they allow visualization of individual features. These models utilize automatic pairwise interaction detection that models the feature function of each target variable and important pairs of features using bagging and gradient boosting, and allow for feature importance to be measured both individually and as group aggregates [24, 26]. As EBMs are a bagged ensemble of trees, they tend to be well-calibrated [27]. We measured feature importance using mean absolute scores on the whole sample and reported area under the receiving operator characteristics (AUROC) on test data for our models predicting 90-day mortality, 90-day readmission, and 1-year revision, and root mean squared error (RMSE) with coefficient of determination (r^2^) for the regression model predicting LOS.

EBMs allow for automatic detection of interaction terms (default by 0.9 times the number of covariates in the model). In a sensitivity analysis, we customized the detection of interaction to allow for the 5 most important interactions. fivefold cross-validation was used to validate prediction accuracy and avoid overfitting for EBM models both with and without interaction. Analyses were performed in Python Jupyter Notebook [28].

## Results

### Cohort characteristics

Our retrospective cohort included 227,959 patients who underwent elective primary TKA. The mean age was 66.3 (SD 9.8) while the median age was 66.0 (IQR [60,73]), and 90.1% of patients were White (Table 1). The median Elixhauser Comorbidity index was 0 (IQR [0,2]). 511 (0.2%) patients died within 90 days, 17,065 (7.5%) patients were readmitted within 90 days, and 1,795 patients (0.8%) underwent revision within 1 year of surgery. The median LOS was 2 days (IQR [2, 3]).

**Table 1:** Characteristics of the cohort

	**Cohort**	**90-Day Readmission**	**90-Day Mortality**	**1-Year Revision**
Variable ^a^	N = 227,959	N = 17,065	N = 511	N = 1795
Age (years)	66.3 (9.8)	69 [62, 67] **	75 [66, 81] **	64 [57, 71] **
**Sex**				
Female	141,063 (61.9%)	10,050 (59.0%) **	251 (49.0%) **	983 (55.0%) **
**Race**				
White	205,364 (90.1%)	15,094 (89.0%) **	448 (88.0%) *	1586 (89.0%) **
Black	14,422 (6.3%)	1456 (8.5%) **	46 (9.0%) *	161 (9.0%) **
Other	7917 (3.5%)	498 (2.9%) **	17 (3.3%) *	43 (2.4%) **
Missing	256 (0.1%)	17 (< 0.1%) **	0 (0.0%) *	5 (0.3%) **
**Discharge Location**				
HHC	57,702 (25.3%)	3652 (21.0%) **	60 (12.0%) **	610 (34.0%) **
Home	27,268 (12.0%)	1497 (8.8.0%) **	17 (3.3%) **	269 (15.0%) **
IRF	11,7982 (51.8%)	8990 (53.0%) **	341 (67.0%) **	644 (36.0%) **
SNF	25,007 (11.0%)	2926 (17.0%) **	93 (18.0%) **	272 (15.0%) **
**Admission Source**				
Clinic or Physician’s Office	31,179 (13.7%)	2140 (13.0%)	85 (17.0%)	178 (9.9%)
Non-Health Care Facility Point of Origin	19,6767 (86.3%)	14,923 (87.0%)	426 (83.0%)	1617 (90.0%)
Other	13 (0.0%)	2 (< 0.1%)	0 (0%)	0 (0%)
**Insurance**				
Commercial	90,965 (39.9%)	4505 (26.0%) **	78 (15.0%) **	733 (41.0%) **
Government	1449 (0.6%)	100 (0.6%) **	6 (1.2%) **	15 (0.8%) **
Medicaid	8780 (3.9%)	871 (5.1%) **	22 (4.3%) **	142 (7.9%) **
Medicare	125,419 (55.0%)	11,514 (67.0%) **	403 (79.0%) **	897 (50.0%) **
Unknown/uninsured	1346 (0.6%)	75 (0.4%) **	2 (0.4%) **	8 (0.4%) **
Diabetes	23,443 (10.3%)	2478 (15.0%) **	76 (15.0%) **	265 (15.0%) **
Hypertension	76,043 (33.4%)	6634 (39.0%) **	175 (34.0%)	820 (46.0%) **
Obesity	31,312 (13.7%)	2856 (17.0%) **	74 (14.0%)	380 (21.0%) **
Elixhauser Comorbidity Index	0 [0, 2]	1 [0, 3]	1 [0, 3]	1 [0, 3]

^a^ Data presented as N (%) or Median [IQR]

^b^ *P*-value calculated via Chi-squared test, Fisher’s exact test, and Wilcoxon rank sum test

^*^ *P*-value < 0.05. ** *P*-value < 0.001. *P*-values compare groups with and without the outcome for all the variables listed above

HHC, health home care; IRF, inpatient rehabilitation facility; SNF, skilled nursing facility

### Patient characteristics by outcome

The median age for 90-day mortality (N = 511) was 75 (IQR [66, 81]). Patients who died within 90 days of surgery were more likely to be men (51%) and discharged to an inpatient rehabilitation facility (IRF) (67%). Patients who were readmitted within 90 days (N = 17,065) had a median age of 69 (IQR [62, 67]). These patients were also more likely to be discharged to an IRF (53%) (*P* ≤ 0.001) and be on Medicare (67.0%) (*P* ≤ 0.001). The median age of patients who underwent revision within 1 year of surgery (N = 1,795) was 64 years (IQR [57, 71]). Patients who underwent revision were more likely to have commercial insurance (41%) (*P* ≤ 0.001), and 46% of these patients had hypertension.

Patients with these adverse outcomes were more likely to have diabetes, hypertension, and obesity, as well as a higher comorbidity burden as measured by the Elixhauser Comorbidity index (median 1 (IQR [0, 3])) (Table 1).

### Prediction model identifies age among most important factors in adverse outcome prediction

Age was among the most important factors and was in the top 5 in all our models with prediction of 90-day mortality, 90-day readmission, 1-year revision, and LOS. The models included the following features: sex, race, discharge location, insurance, and comorbidities.

**Fig. 1:**
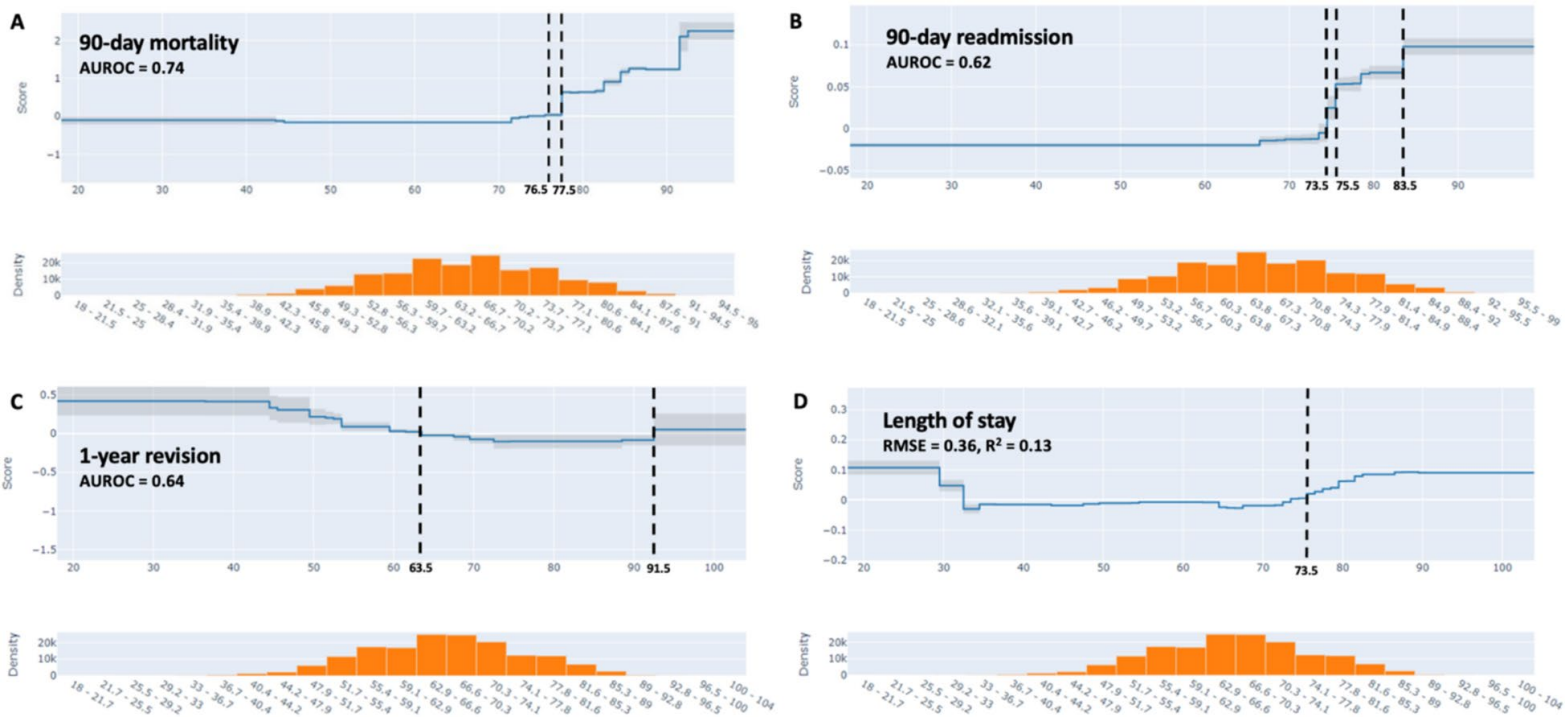
Partial dependence of age (years) on (**A**) 90-day mortality, (**B**) 90-day readmission, (**C**) 1-year revision, and (**D**) length of stay. Y-axis score in log scale. Vertical lines demonstrate where the risk score is equal to 0, and show the average contribution of age to risk

The AUROC was 0.74 for the prediction of 90-day mortality, 0.62 for 90-day readmission, and 0.64 for 1-year revision. The prediction model for LOS had an RMSE of 0.36 and an R^2^ of 0.13 (Fig. 1). The fivefold cross-validated AUROC was 0.74 (SD = 0.03) for 90-day mortality, 0.63 (SD = 0.00) for 90-day readmission, and 0.64 (SD = 0.02) for 1-year revision. The cross-validated RMSE was 0.36 (SD = 0.001) for LOS.

### Relationship of age and TKA outcomes

The EBM model allows us to understand as well as visualize the relationship between age and TKA outcomes, accounting for all of the other features studied, including comorbidities. The predicted risk for 90-day mortality increased at 76.5 years, and again substantially at 77.5 years. The risk of 90-day readmission increased at 73.5 years, and there was a steep rise again at 75.5 and 83.5 years, indicative of increases in risk. The risk of 1-year revision, however, was greater before 63.5 years and after 91.5 years (very low volume of surgeries above 89 years). The risk for longer LOS increased at 73.5 years (Fig. 1). In a secondary analysis, we trained EBM models allowing up to 5 two-way interaction predictors. The AUROCs were similar to models without interactions.

**Fig. 2:**
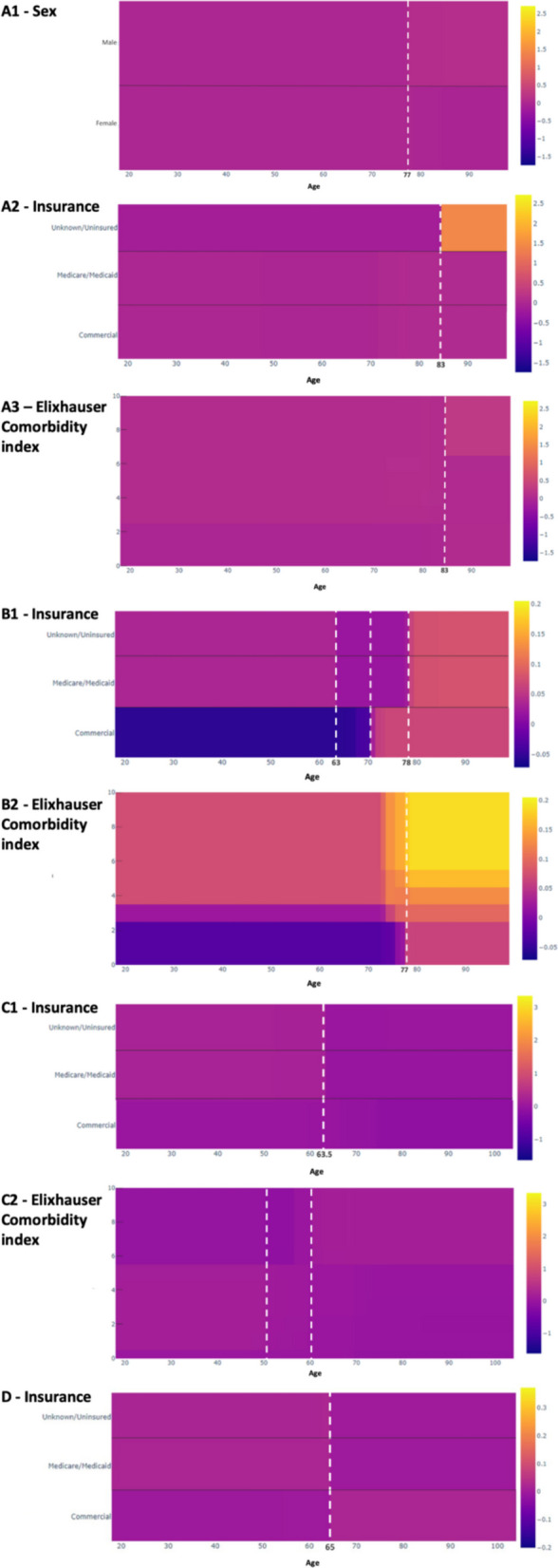
Partial dependency plot of (**A**) 90-day mortality, (**B**) 90-day readmission, (**C**) 1-year revision, and (**D**) LOS and the interaction of age (years, x-axis) with predictive factors (y-axis). Predictive factors include: (A1) patient sex, (A2) insurance, (A3) Elixhauser Comorbidity index, (B1) insurance, (B2) Elixhauser Comorbidity index, (C1) insurance, (C2) Elixhauser Comorbidity index, and (D) insurance. Color corresponds to the mean absolute score, with darker color demonstrating a more negative score and lighter color demonstrating a more positive score (see right-hand axis)

Patient sex, insurance, and Elixhauser influenced the relationship between age and 90-day mortality (Fig. 2A). Mortality risk increased for men above the age of 77, but did not change for women. At 83 years, patients with unknown/uninsured status demonstrated a substantial increase, and patients with Elixhauser scores > 6.5 demonstrated a mild increase in mortality risk.

The relationship of age and 90-day readmission was influenced by insurance and Elixhauser (Fig. 2B). Uninsured or unknown insurance and Medicare/Medicaid patients had a higher risk below the age of 63 compared to 63 to 78 years, after which point risk increased substantially. This may indicate that the optimal age for patients with insurance other than commercial may be from 63 to 78 for the 90-day readmission risk. Conversely, the risk of 90-day readmission in patients with commercial insurance increased at 70 years. Risk increased at 77 years regardless of Elixhauser, but increased the most for patients with Elixhauser scores > 6. The presence of diabetes was another interaction that increased readmission risk.

Insurance and Elixhauser influenced the relationship of age and 1-year revision (Fig. 2C). Revision risk increases across each examined insurance before 63.5 years, most prominently in uninsured or unknown insurance and Medicare/Medicaid patients. Irrespective of comorbidities, the risk of 1-year revision decreases at 50 years, but in those with Elixhauser scores < 5.5, the risk further decreases at 60 years.

In the examination of age and LOS, only insurance influenced this relationship (Fig. 2D). LOS risk increases in patients with commercial insurance and decreases in patients with all other examined insurance after 65 years. Race, diabetes, hypertension, and obesity were included in the examination of age and adverse TKA outcomes, but these factors, as well as their interactions with age, were not among the top factors that influenced the relationship of age and outcomes.

## Discussion

In our analysis of more than 227,000 patients who underwent TKA, we determined that the effect of age as a risk factor for poor TKA outcomes changed at specific time points, thus demonstrating that there is a nonlinear relationship between age and TKA outcomes. Even after accounting for other demographics and comorbidities, our data suggests that the optimal age for lower risk of 90-day mortality, 90-day readmission, and longer LOS is below 73.5 years, and above 63.5 years for 1-year revision. When investigating factors that influence the relationship of age and TKA outcomes, we found that mortality increased specifically for males above 77 years, and that those patients with non-commercial insurance had a lower risk of readmission from 63 to 78 years. Our work demonstrates a complex relationship of age and TKA outcomes, and can help in decision-making when trying to quantify risks in this procedure, which is progressively used across all ages.

A detailed systematic review to find the optimal age for TKA suggested that patient-reported outcomes, mortality, and revision risk were good between the ages of 70 and 80 years, and best around 70 years [29]. While our data is somewhat similar, the glass box machine learning model helps us visualize the nuances and shows the optimal age to be between 63.5 and 73.5 years, thus including younger patients. Previous studies have used machine learning to understand TKA outcomes with prediction performance (AUROCs) comparable to ours, but have not taken a deeper dive into understanding age as a risk factor and its relationship with other patient-level factors [30–32]. One such study identified age as an important factor in the prediction of complications within 90 days of surgery, similar to the findings in our study [32]. However, we further provide cutoff points where the risks change substantially. By providing insight into the specific changes in risk across the patient lifespan, these findings may inform the ages at which patients and clinicians consider and elect TKA by providing a framework guide for risk stratification. Notably, our models included known patient-level risk factors such as race and individual comorbidities (diabetes, hypertension, and obesity) [21–23, 33], however these were not among the top interactions in the prediction.

While only 0.2% of our population died within 90 days of TKA, we found that mortality increased at 76.5 years, and again substantially at 77.5 years. Our findings are consistent with reports of increased mortality with age [9, 11], which could be a function of multiple patient, hospital, or policy-level factors. While men are known to have an increased risk of mortality after TKA, we observed that this risk increases substantially at the age of 77 [9, 34]. Our data also showed substantially elevated risk of mortality in uninsured or unknown insurance patients above the age of 83, suggesting that these TKA operations may have happened due to extreme operative need.

A total of 7.5% of our population was readmitted within 90 days, and our findings were similar to prior studies, where risk increased with age [35, 36]. Further, our data demonstrated that risk increased after the age of 73.5 years and at certain time points, including 75.5 and 83.5 years. Our data support the known literature that comorbidities increase the risk of readmission [37]. Risk increased in patients with commercial insurance at 70 years, which we hypothesize may be the result of a high rate of return to work post-surgery. Conversely, uninsured and Medicare/Medicaid patients were at decreased risk of 90-day readmission between 63 and 78 years, likely reflecting better insurance coverage due to eligibility for Medicare at 65.

Our model predicted an increased risk of 1-year revision in patients before the age of 63.5, since younger patients may have higher levels of physical activity or sport injuries/trauma [38, 39]. Further, patients with increased comorbidities were at higher risk after 55.5 years, given their association with increased complications [40]. Medicare/Medicaid and uninsured or unknown insurance patients were at especially greater revision risk below the age of 63, and increased risk of longer LOS above the age of 65. These findings reflect the role of policies and insurance eligibility, which change at the age of 65, and in a way validate our model, which is artificially influenced by Medicare eligibility.

Our study is not without limitations. We only included patients who underwent primary elective TKA in Pennsylvania, and our results, therefore, may not apply to other regions of the US. Secondly, our models have limited predictive power (AUROCs 0.62, 0.64, and 0.74 for 90-day readmission, 1-year revision, and 90-day mortality, respectively); they were comparable to other administrative studies of arthroplasty outcomes [30–32, 41]. As with many flexible machine learning models, EBMs are susceptible to overfitting. To prevent this, we allowed up to 5 two-way interaction terms. Lastly, although we identified ages where the risk of adverse TKA outcomes is predicted to increase or decrease, many other factors may influence these ages. This may include variables previously shown to be important in TKA outcomes, such as surgeon or facility volume or rural vs. urban location [35, 42, 43], as well as other unidentified system- or hospital-related factors.

There are many strengths to our study. The size and breadth of our cohort is a great advantage, including 227,959 patients with associated patient-level data. Further, we utilized the glass box EBM models, which show great promise as they account for nonlinear relationships and interactions, and are much more flexible than parametric models such as logistic regression models [44]. Traditional regression models have always been understood to have a proportionate increase in risk as age increases. However, utilization of EBMs allowed us to address nonlinear relationships and accurately assess the risk of adverse TKA outcomes as they change over the patient’s lifespan. Our study demonstrates the efficacy of EBM models at a population-based level and supports the future application of machine learning to national and international datasets. Prior work at the international scale demonstrated differences in TKA outcomes, such as revision and mortality, between the US and Canada [45], and EBMs may provide greater insight into the factors that influence risk of these outcomes and demonstrate differing age cutoffs in different populations. The extensive data in these larger datasets may improve the applicability of our prediction models and facilitate the development of precision medicine tools that can provide individual risk scores.

## Conclusions

In summary, using a glass box supervised machine learning model we were able to demonstrate that risk for poor TKA outcomes significantly changes with age at various time points. Our study provides a framework for risk assessment on a population level and suggests that the optimal age to undergo TKA is below 73.5 years for a lower risk of mortality, readmission, and longer LOS, and above 63.5 years for 1-year revision. Particularly, the risk of mortality increased after 77 years in males, and the risk of readmission decreased in patients with non-commercial insurance between 63 and 78 years. Therefore, understanding the nuance of age and its interactions with other factors in TKA risk assessment is important for planning and decision-making for all stakeholders, and further studies would be helpful to design precision medicine tools to predict risk scores for individual patients.

## Data Availability

The dataset analysed during the current study is available from the Pennsylvania Health Care Cost Containment Council, https://www.phc4.org.
